# Pyridine as an additive to improve the deposition of continuous electrospun filaments

**DOI:** 10.1371/journal.pone.0214419

**Published:** 2019-04-25

**Authors:** Antonina A. Lach, Hayley L. Morris, Joana A. Martins, Edward T. Stace, Andrew J. Carr, Pierre-Alexis Mouthuy

**Affiliations:** 1 Botnar Institute of Musculoskeletal Sciences, Nuffield Department of Orthopaedics, Rheumatology and Musculoskeletal Sciences, University of Oxford, Oxford, United Kingdom; 2 NIHR Oxford Biomedical Research Centre, John Radcliffe Hospital, Oxford, United Kingdom; University of Lisbon, PORTUGAL

## Abstract

Electrospun filaments are leading to a new generation of medical yarns that have the ability to enhance tissue healing through their biophysical cues. We have recently developed a technology to fabricate continuous electrospun filaments by depositing the submicron fibres onto a thin wire. Here we investigate the influence of pyridine on the fibre deposition. We have added pyridine to polydioxanone solutions at concentrations ranging from 0 to 100 ppm, increasing the conductivity of the solutions almost linearly from 0.04 uS/cm to 7 uS/cm. Following electrospinning, this led to deposition length increasing from 1 cm to 14 cm. The samples containing pyridine easily underwent cold drawing. The strength of drawn filaments increased from 0.8 N to 1.5 N and this corresponded to a decrease in fibre diameter, with values dropping from 2.7 μm to 1 μm. Overall, these findings are useful to increase the reliability of the manufacturing process of continuous electrospun filaments and to vary their biophysical properties required for their application as medical yarns such as surgical sutures.

## Introduction

Surgical sutures are one of the most successful biomaterials-based medical devices used today, with a market currently exceeding $1.3 billion (USD) annually [1]. Sutures are designed to mechanically support the closure of a wound or incision. Most modern sutures are made of synthetic polymers that are either degradable (e.g. polydioxanone—PDO) or non-degradable (e.g. polypropylene) [1–6].

Melt spinning and extrusion are typically used for processing synthetic polymers into filaments. The extrusion process is followed by a drawing step to stretch filaments up to several times their initial length before being further processed to create the suture material [7, 8].

Although mechanical performance has always been a primary focus, the repair of some tissues could benefit from sutures that also actively contribute to the healing process. This is particularly important for tissues with poor repair ability such as tendon and ligaments [9–14].

In the search for new suture strategies, electrospun materials made of submicron fibres have been proposed to provide biophysical cues to cells in the wound area [15–17]. Electrospinning uses electrical charges to produce submicron fibres that mimic the extracellular environment [18]. Although electrospun materials have been mostly produced in the form of non-woven meshes, approaches to producing long continuous filaments have been reported. Continuous filament production utilizes modified collection methods such as a progressing belt [19, 20], funnel [21–23] or a water bath [21, 24–26].

We have recently reported a method which enables the fabrication of continuous electrospun PDO filaments using a thin wire as a collector [16, 27, 28]. Once deposited, the filaments are detached and further processed before use. A cold drawing step is typically applied to prevent filament from deformation during its further use. The drawing also aligns the submicron fibres in the direction of the filament and improves its mechanical properties. The stretched electrospun filaments are the single units that can be assembled into twisted or braided multifilament yarns [16, 29]. *In-vivo* studies showed that PDO electrospun multifilament yarns can be safely used as suture materials for tendon repair [16].

To ensure the viability and robustness of our previously established filament fabrication technology, in particular in the context of industrial scale-up, control over the fibre deposition is essential. Compared to traditional collectors such as plates and drums, the collection surface offered by the wire is relatively small. As a result, the deposition is more sensitive to slight perturbations of the electrical field such as by the presence of static charges on the walls of the spinning chamber. While experimenting with the method, we have observed that the deposition length of the fibres on the wire (length of the static wire covered with fibres) was crucial to the quality of the filament produced. In particular, for a given polymer solution, larger deposition lengths typically resulted in better quality filaments (i.e. filaments which can undergo post-fabrication process, such as drawing, without breaking). To achieve consistently wide deposition and higher yield of good quality filaments, our strategy was therefore to increase the conductivity of the polymer solutions. Positive outcomes using this approach have already been observed by our research group in previous experiments, and it is known to lead to larger deposition area on two-dimensional collectors (e.g. plates) (unpublished data). In this study we have selected pyridine in order to modulate polymer solution’s conductivity, which is easily available, an FDA approved compound and has already been used as an additive in the pharmaceutical, agricultural or food industries [30–33].

Pyridine has previously been used in the electrospinning field as a means to increase polymer solution conductivity and spin ultrafine electrospun fibres [34, 35].

The objective of this study was to investigate whether pyridine could improve the reliability of electrospinning process of PDO filaments on the wire collector. Our first hypothesis was that the addition of pyridine to the polymer solution would increase its conductivity, leading to larger deposition lengths on the wire, and therefore significantly increase the ratio of better filament quality. Our second hypothesis was that pyridine would change the morphology and mechanical properties of the filaments. Characterisation methods applied in this study included observations of the spinning process as well as morphological and mechanical evaluations of electrospun fibres and filaments, prior to and after drawing.

## Materials and methods

### Preparation of the polymer solutions

Polydioxanone (PDO, melting flow rate 5.1g/10 min and 5.6g/10 min at 170°C, Riverpoint Medical, Portland, USA) was dissolved in 1,1,1,3,3,3-hexafluoroisopropanol (HFIP, ultra-pure >99.99%, Halocarbon Product Corporation, Atlanta, USA) at the concentration of 7% (w/v) with the addition of pyridine (purity >99.5%, PhEur grade, EMSURE ACS, Merck, Darmstadt, Germany). Pyridine was added to the polymer granules along with HFIP at different concentrations: 0 ppm (control), 1 ppm, 5 ppm, 10 ppm, 50 ppm, 100 ppm. Prior to further testing, the final solutions were agitated at room temperature on a roller for at least 72 hours to allow for complete dissolution of the polymer granules.

### Viscosity of the polymer solutions

The dynamic viscosity of polymer solutions was measured using a rotational viscometer Viscopad R and TR8 spindle (Fungilab, Barcelona, Spain). Prior to measurements, polymer solutions were mixed to ensure their homogeneity. A volume of 7.1 ml was poured into a stainless steel container and the spindle was submerged in the polymer solution. All tests were carried out in a water bath at 18°C resulting in temperature variations of less than 0.8°C. The viscosity measurements were carried out with a spindle speed of 20 RPM. Readings of the dynamic viscosity were taken after 5 minutes from the start of the experiment to allow polymer solution’s flow to stabilise. The tests were performed in three repeats for each concentration.

### Conductivity of the polymer solutions

The solution conductivity was measured using the bench top conductivity meter SevenCompact S230 and InLab 741 ISM probe (Mettler Toledo, Leicester, UK). Prior to measurements, polymer solutions were mixed to ensure their homogeneity. The probe was immersed in solutions and conductivity values (μS/cm) were read from the meter’s screen along with temperature (°C). All tests were performed in three repeats for each concentration.

### Fabrication of continuous electrospun filaments

**Fig 1:**
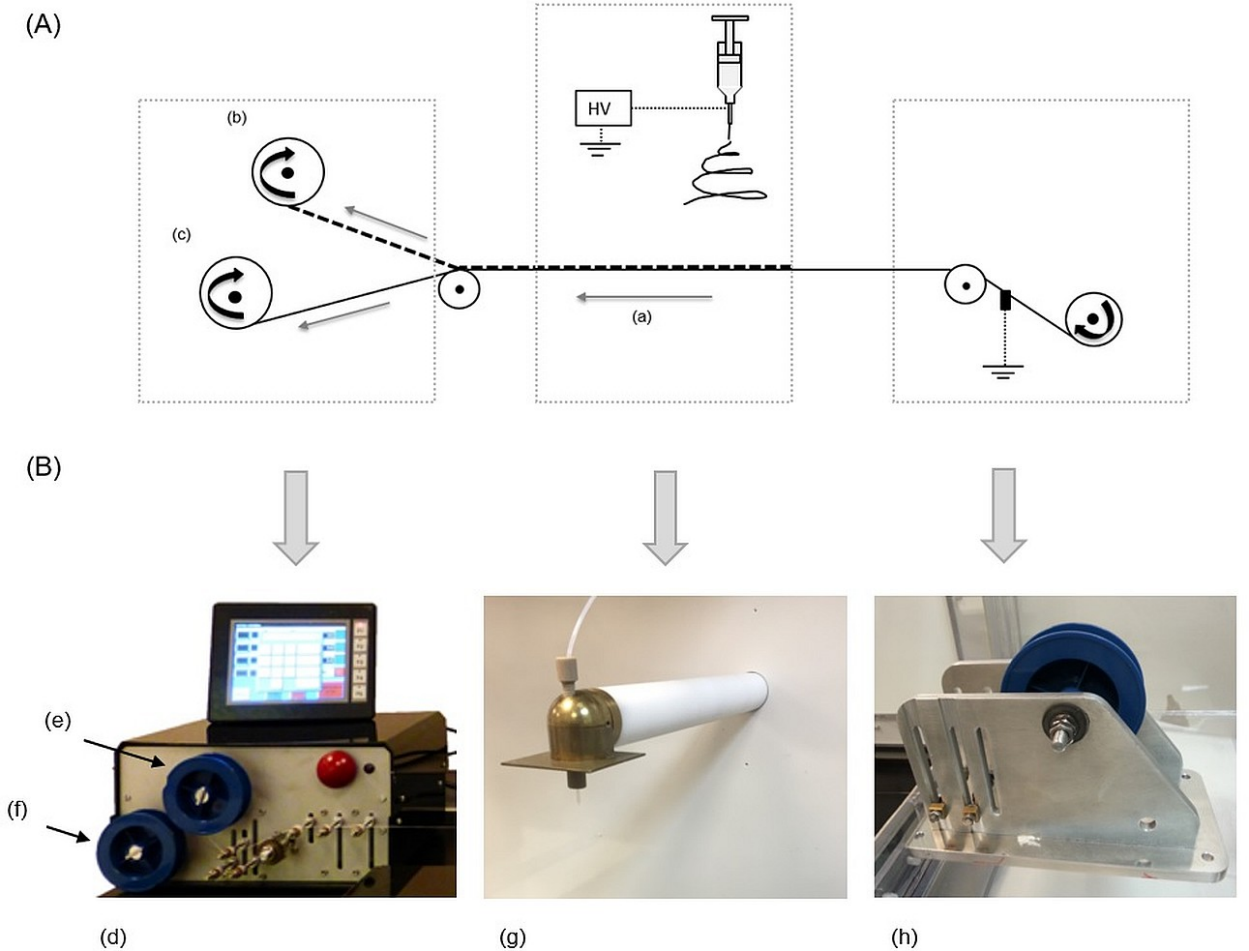
Electrospinning setup used in this study. (A) schematic of the process for the fabrication of continuous filament [16]; (a) direction of the wire movement, (b) filament collecting spool, (c) wire collecting spool; (B) actual equipment—various components installed inside a glovebox: (d) software-controlled collecting unit with spools for the freshly detached filament (e) and for the progressing wire (f), brass nozzle mounted inside the glovebox (g) with wire underneath (not visible in the picture), wire feeding unit (h).

The electrospinning of continuous filaments was carried out using a thin grounded wire as a collector according to a method described previously [16] inside a glovebox and using the setup presented in Fig 1. Briefly, we have utilized a single nozzle electrospinning setup with a high voltage power supply system (up to 30kV, SL30P30/230, Spellman, West Sussex, UK) and a syringe pump (World Precision Instruments Limited, FL, US). The stainless steel wire (100 μm in diameter, Goodfellow, Huntingdon, UK) was moved linearly underneath the charged nozzle at a speed of about 0.5 mm/s with a bespoke collecting unit. The distance between the nozzle and the collector was set at 20 cm. The flow rate applied was 0.8 ml/h, and average voltage applied was 9.2 kV, however the voltage varied between experiments to obtain stable spinning conditions. The electrospun filaments were collected on a spool, which was installed on the collecting unit. Since the wire was moving underneath the nozzle continuously, the length of filament produced was defined by spinning duration. After fabrication, filaments were stored in a desiccator under low humidity and at room temperature until further use. These experiments were performed in three repeats by three different operators, on different days, defining 3 batches (n = 3). The same stock of polymer solution was used for filament fabrication.

### Evaluation of the spinning process

For each filament batch preparation, several observations were recorded. These included the jet length (length of the straight jet path directly following the Taylor cone, which was assessed using a ruler positioned outside of the glovebox), the ability to detach the filament from the wire and the outcome of electrospinning.

Although the bespoke collecting unit allows the detachment and winding of filaments onto the filament collecting spool during the fabrication, in our experiments this step was done manually. In order to assess the ability to detach filaments from the collecting wire, filaments were manually removed from the wire and the ability to detach was graded on a scale of easy (no resistance to detachment), medium (resistance but no damage caused to the filament) or difficult (detachment not possible or major damage caused to the filament). For each of the experiments, the operators recorded whether the filament was produced or not, as well as the ability to use the filament for further processing (drawing in particular).

### Deposition length verification

The deposition length was assessed in a separate set of electrospinning experiments, carried out from 3 stocks of PDO polymer solutions on different days by one operator. The same electrospinning setup was used, however in this case, the wire collector was kept static (collecting unit was turned off). The filaments were spun with the flow rate 0.8 ml/h for 1 minute and the average voltage applied was 12.0 kV, however voltage was adjusted in each experiment to enable jet stabilization and electrospinning. The electrospun material was kept on the wire for assessment of the deposition length on the wire (i.e. length of the wire visibly covered with electrospun fibres).

### Defining the drawing/stretching ratio

The drawing ratios (DRs), or stretching ratios, of the electrospun filaments were determined for each concentration of pyridine. To do this, filaments were first stretched using Zwick tensile machine (Zwick Roell Group, Ulm, Germany). Experiments were carried out at the rate of 25 mm/min until failure. The distance between grips was set at 50 mm and the force at break (N) and elongation (mm) were recorded. Tests were carried out in 5 replicates per each batch and TestXpert software was used to collect data and to generate force-elongation graphs.

While stretching, filaments underwent two sub-processes: plastic and elastic deformation, which were reflected on force-elongation graphs. In order to determine the Transition Point (TP) between them, we analysed individual graphs using the method described in S2A and S2B Fig. In brief, graphs were bisected into two linear regions representing each sub-process. Two lines converged at a point which indicated the transition between two types of filament behavior, defining the TP. The assessment of each graph was performed by three operators independently and average TP for each pyridine concentration was calculated.

DR was based on the average strain at break (mm) calculated for each concentration of pyridine. We defined the DR as the drawing ratio achieving 75% of the elongation between the TP and maximum strain at break (filament’s break point). This drawing ratio was selected after testing different ratios and was set to ensure that the average drawn filament would be fully stretched (beyond the TP) without breaking.

### Drawing

Consistent and smooth filament sections (10 cm) were placed on a ruler (resolution 1.0 mm) and manually stretched using the stretching ratio calculated for each individual batch. A similar speed of elongation was applied to all samples, and drawing was performed by one operator. Five filaments were stretched per batch and drawing was performed at room temperature. After manual stretching, the filaments were stored in a desiccator under low humidity and at room temperature until further use.

### Tensile properties

The filaments (prior to and following manual drawing) were mechanically characterized by analyzing five samples from each batch in both filament groups using Zwick tensile machine (Zwick Roell Group, Ulm, Germany). The tests were carried out at the rate of 25 mm/min until failure. The distance between grips was set at 50 mm. The force at break (N) and rate of elongation (mm) were recorded. The TestXpert software was used to collect data.

### Scanning Electron Microscopy (SEM)

The electrospun filaments were imaged by scanning electron microscopy (SEM, Zeiss, EVO LS15, Oberkochen, Germany). Samples were cut from randomly selected sections of the filaments and mounted on aluminum stubs using carbon adhesive tape. They were then gold/palladium coated for 120 seconds using a sputter coater system (SC7620 Mini Sputter Coater, Quorum Technologies, Laughton, UK). Images were taken at magnifications 5000x, 7500x and 10.000x. The fibre diameters were measured using ImageJ image analysis software (W.S. Rasband, National Institutes of Health, Bethesda, USA).

### Statistical analysis

Statistical analysis was performed with GraphPad Prism 7 software. Data are expressed as means with standard deviations. To determine the statistical significance of results, a standard one-way ANOVA test followed by Tukey test, and a Correlation test were performed. Results were considered significant at p<0.05.

## Results

### Solution properties: Viscosity and conductivity

**Fig 2:**
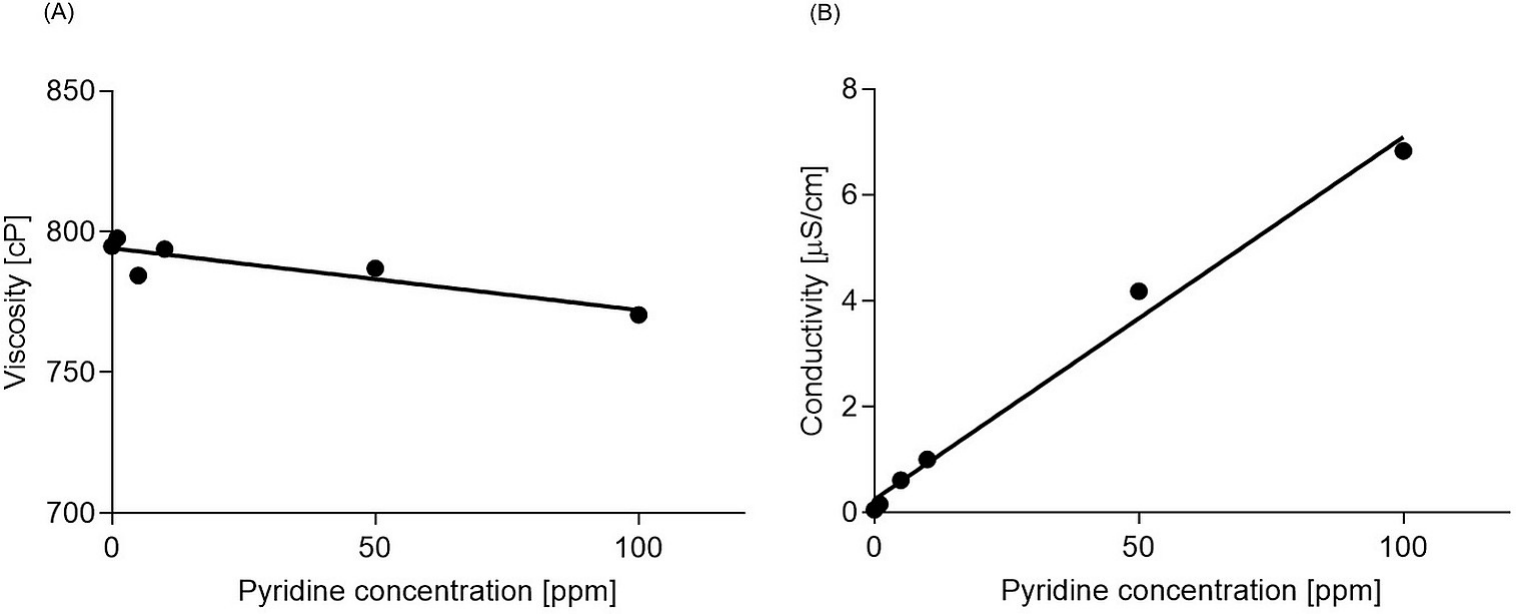
Properties of PDO solutions (7% w/v PDO in HFIP) containing different concentrations of pyridine. (A) Dynamic viscosity (n = 3) (r = - 0.88, R^2^ = 0.78, p = 0.0195). (B) Conductivity (n = 3) (r = 1.0, R^2^ = 0.99, p<0.0001). Abbreviations: PDO–polydioxanone, HFIP–hexafluoroisopropanol, w/v–weight to volume ratio.

The polymer solutions were tested for their dynamic viscosity and conductivity. Pyridine decreased the dynamic viscosity of the polymer solution as shown in Fig 2A, and led to an almost linear increase in its conductivity as shown in Fig 2B. Correlation coefficient was used for statistical analysis.

### Observations of the electrospinning process and its outcome

The observations of the electrospinning process (Table 1) have shown that with the absence of pyridine, the straight jet was relatively long, being around 8 cm on average. In the presence of pyridine, the jet became increasingly shorter as the concentration of pyridine was increased, reducing the jet length to approximately 1–2 cm at 100 ppm.

**Table 1:** Detailed observations of individual electrospinning experiments and samples produced with different concentration of pyridine (Py). Filament fabrication was carried out in 3 repeats (n = 3), deposition length was assessed in separate set of experiments (electrospinning onto static wire) in three replicates (N = 3). Abbreviations: N/A–not applicable.

Py [ppm]	Voltage [kV]	Jet length [cm]	Deposition length [cm]	Filament detachment	Filament usability
0	7.3–10.9	7–9	0.8	N/A or easy	No filament or usable filament
1	8.2–9.4	5–8	5.8	easy	Usable filament
5	8.4	3–5	7.7	easy	Usable filament
10	8.4–10.4	2–3	9.8	medium	Usable filament
50	8.6–10.7	1–2	13.3	medium	Usable filament
100	8.6–10.7	1–2	14.2	medium	Usable filament

**Fig 3:**
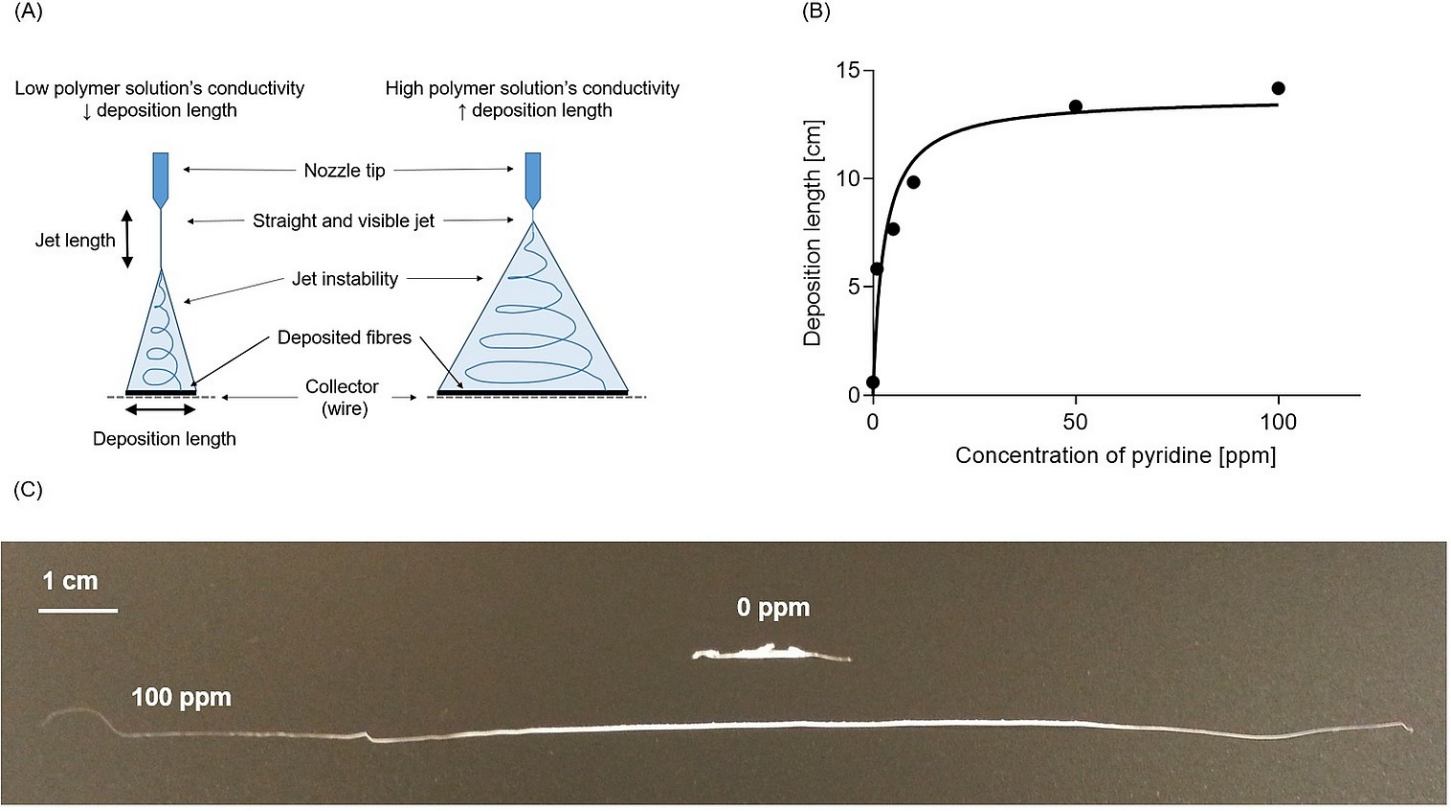
Deposition length. (A) Schematic depiction of mechanisms governing deposition length; (B) Average deposition length (cm) for the control (0 ppm) and solutions with different pyridine concentrations. Experiment (electrospinning onto a static wire) was run in three replications (N = 3) (Nonlinear regression analysis was used, R^2^ = 0.94); (C) Examples of detached filament obtained after 1 minute of electrospinning onto the static wire for the 0 ppm and 100 ppm polymer solutions.

Fig 3A explains the mechanisms governing the deposition length. The deposition length (experiments with the static wire collector) obtained from the control solution was below 1 cm and increased progressively across all concentrations, from 6 cm at 1 ppm up to 14 cm at 100 ppm (Fig 3B). The significant increase in deposition length (One-way ANOVA, p<0.0001) resulted in usable filaments in all pyridine concentrations (as opposed to the control solution). It is worth noting that this increase often led to a more flat and smooth deposition profile (the material was better spread around and along the wire). Examples of filaments fabricated from the control solution (0 ppm pyridine) and from the solution with 100 ppm of pyridine are shown in Fig 3C. The presence of pyridine in polymer solutions clearly improved filament fabrication (experiments with the progressing wire) and increased the yield of usable filaments produced, as filaments were successfully spun in all attempts from solutions containing pyridine. Although the filaments occasionally presented faults such as peaks, all filaments produced with pyridine were able to undergo drawing and handling. Without pyridine, it was difficult to produce filaments that could undergo post-processing (only one experimenter out of three managed to produce usable filament).

### Drawing ratio and tensile properties

**Fig 4:**
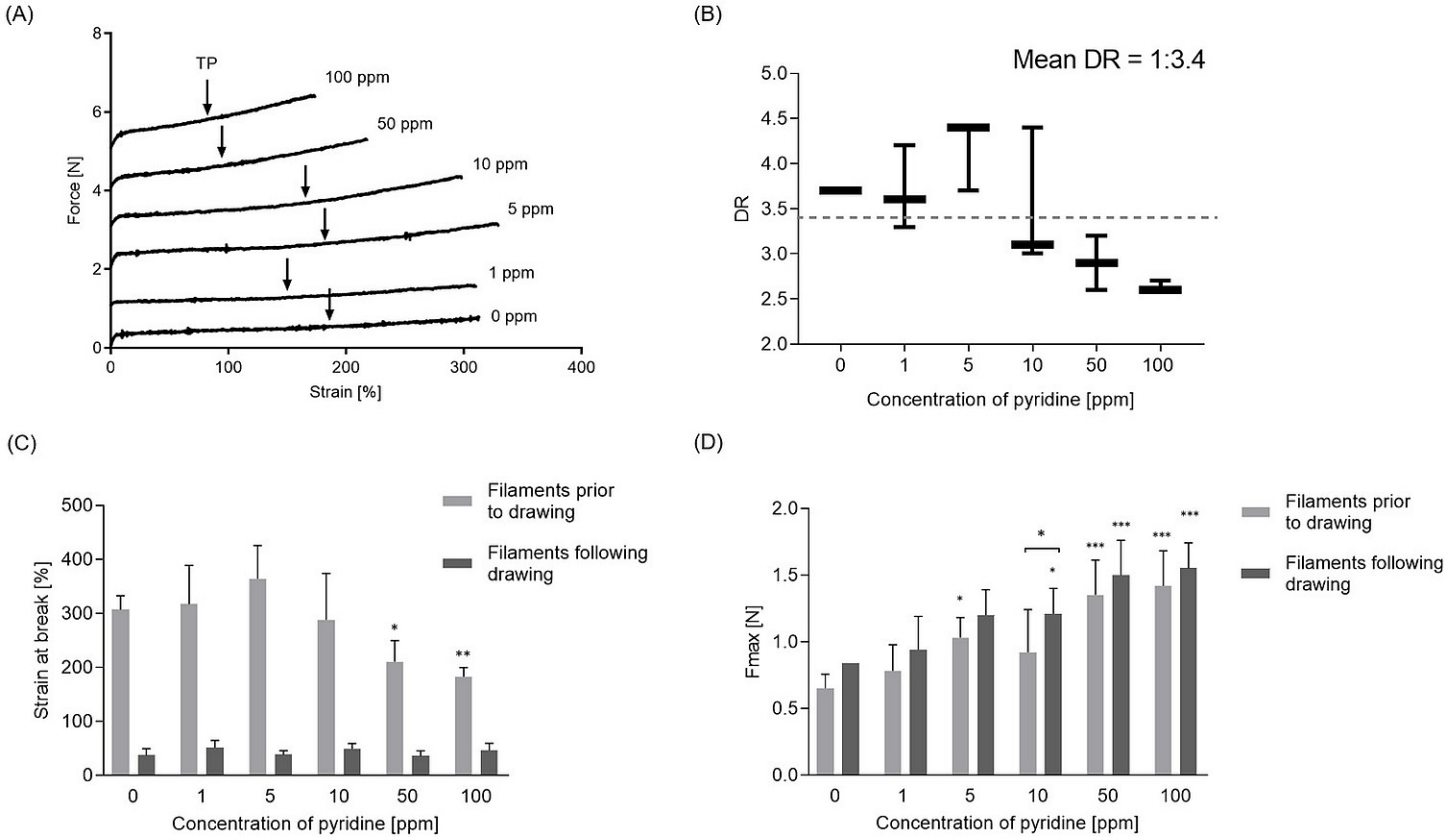
Mechanical properties of electrospun filaments prior to drawing and following drawing. (A) Force (N)—Strain (%) curves obtained in stretching experiment. Stretching behavior of representative filament spun from polymer solution containing different concentration of pyridine. Arrows indicate Transition Point (TP) between plastic and elastic deformation. Note: in order to present six different curves on one graph, the force values for all samples apart from 0 ppm have been translated and values of 1 N, 2 N, 3 N, 4 N and 5 N were added to 1 ppm, 5 ppm, 10 ppm, 50 ppm and 100 ppm curves respectively; (B) Values of the DRs defined for each pyridine concentration. DR was established upon testing 5 filaments sections obtained from each three filaments batches per each concentration of pyridine; (C) Strain at break (%) of filaments prior to and following drawing; (D) Fmax (N) for filaments prior to and following drawing. Note: abbreviation: Fmax–maximal force, force at break; DR–drawing ratio; TP–transition point. For (A), (B) and (C): N = 3, n = 5. Error bars represent standard deviation.

The different concentrations of pyridine governed the drawing ratios (DRs) and the tensile properties of the filament (Fig 4). The DRs were established according to the method described in ‘Defining the drawing/stretching ratio’ section in ‘Materials and Methods’ and in S2A and S2B Fig. The average DR for all filament batches assessed in this study is 1:3.4 (meaning an average increase in length of 3.4 times). One-way ANOVA followed with Tukey’s multiple comparison test were used for statistical analysis. When compared to the control, the change in DRs was not statistically significant for all pyridine concentrations. Interestingly, the cold drawing of electrospun filaments was accompanied by a “double neck” formation at both the macro-level (filament) and micro-level (individual fibre) (S2C Fig).

The statistical significance of strain at break was calculated to compare both groups of filaments (prior to and following drawing) and within each group of filaments (strain of filament with each pyridine concentration was compared to the control filament). As expected, the strain at break of filaments prior to drawing was significantly higher (p<0.0001) than strain at break of drawn filaments across all pyridine concentrations (Fig 4C). The strain at break values of the filaments prior to drawing follow a trend similar to the drawing ratios presented in Fig 4B. In ‘prior to drawing’ filament group the strain at break was statistically different for pyridine concentrations 50 ppm (p = 0.0252) and 100 ppm (p = 0.0014), when compared to 0 ppm filaments.

The presence of pyridine in PDO polymer solution improved the strength of both filaments groups (before and after drawing), when compared to the control (0 ppm) (Fig 4D). Statistically significant increases in strength were observed in 5 ppm filaments before drawing (p = 0.0365), and in both groups in 50 ppm and 100 ppm concentrations (p<0.0001). The drawing step seemed to further increase the filament strength, however this change was only significant for 10 ppm group (p = 0.0441).

### Diameter of the fibres in the electrospun filaments

**Fig 5:**
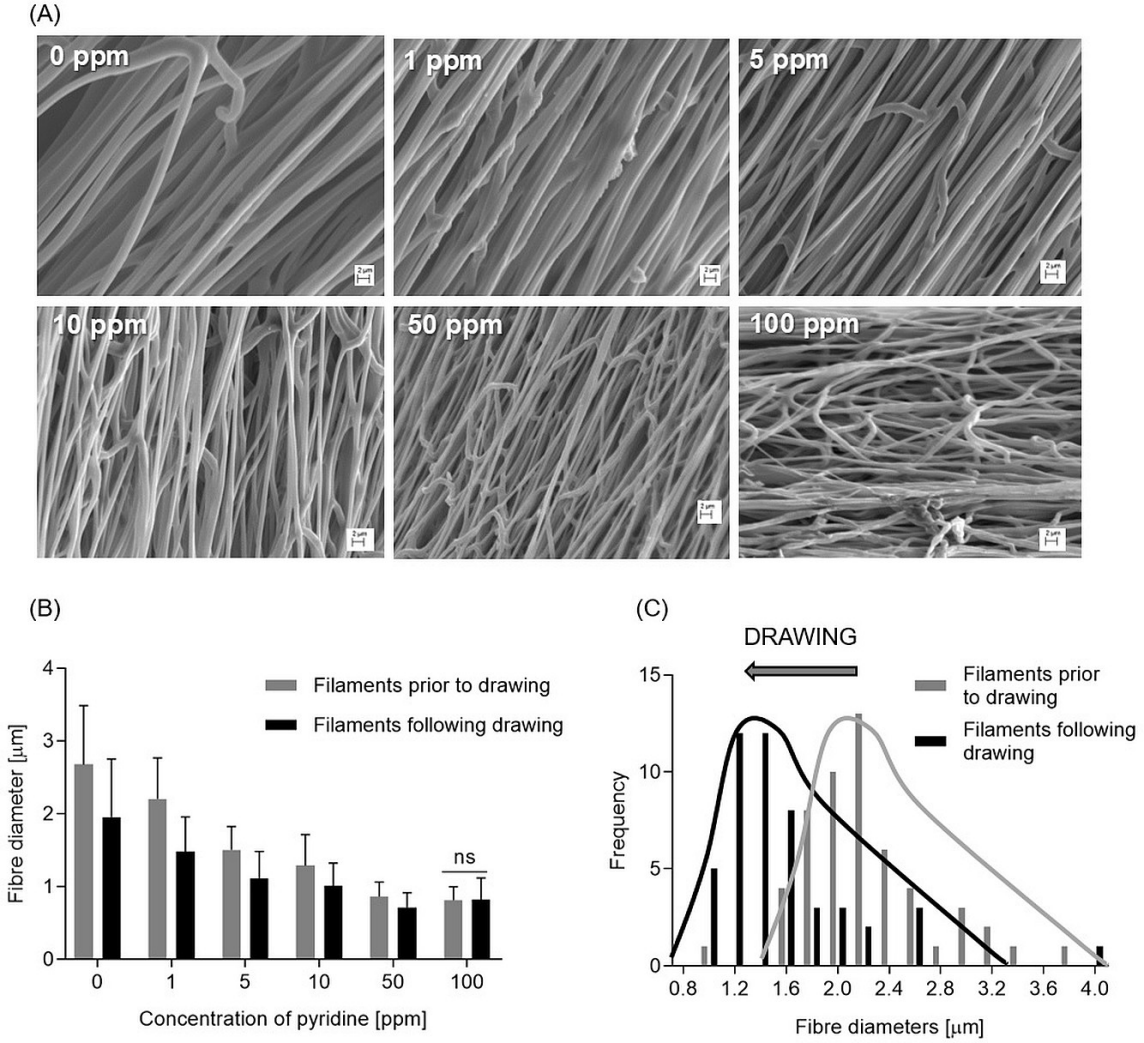
Morphology of the submicron fibers in the drawn electrospun filaments. (A) Fibre diameter measured in the filaments (prior to and following drawing) produced from polymer solutions with different concentration of pyridine. The average fibre diameters are obtained from three batches of filaments (n = 3). Error bars represent standard deviation; (B) Change in distribution of fibre diameter due to drawing the filament with 1 ppm pyridine. 0 ppm, 5 ppm, 10 ppm and 50 ppm showed a similar change. (C) SEM images of the electrospun fibres in the drawn filaments (magnification 5000x).

Fibre diameter of filaments before and after drawing significantly decreased with increasing concentrations of pyridine (Fig 5A, p<0.0001). Fig 5A and 5B also show the effect of drawing, which led to a reduced average fibre diameter by about 25% on average (for pyridine concentrations 0 ppm– 50 ppm). This reduction in fibre diameter was statistically significant (p<0.0001) for all concentrations apart from 100 ppm.

## Discussion

The electrospinning process can be tuned by a number of variables including the polymer solution properties, processing parameters, and environmental parameters [36]. The aim of our investigation was to improve the stability of electrospun filament manufacture. This was achieved by modulating the conductivity of the stock polymer solution through the addition of pyridine. The resulting effect was an increase of the electrospun fibre deposition length on the wire collector. We believe that larger deposition length could improve the reliability of the process and result in high yield of the good quality filaments. The results from our investigation could be applied in the context of technology scale-up and could be extended to other polymers.

### Low concentrations of pyridine increase the conductivity of polymer solutions

To investigate the relationship between pyridine concentration and the polymer solution properties, we have added pyridine to PDO solutions at concentrations ranging from 1 ppm to 100 ppm. This range was much lower than the concentrations that have been looked at so far by other researchers in the context of electrospinning. Moghe [37] worked with polycaprolactone in glacial acetic acid and pyridine above 1000 ppm, Zeng [38] investigated the addition of pyridinium formiate to PLA/dichloromethane solutions at 2000–8000 ppm, Huang [34] worked with nylon-4,6 dissolved in formic acid and investigated the effect of pyridine in concentrations between 370 and 4000 ppm by weight.

Our study showed significant decrease in polymer solution’s viscosity when increasing the pyridine concentration, however the increase in the conductivity across the same pyridine concentrations was more explicit, with a trend which is almost linear. Despite the fact that we looked at a lower range of concentrations (below 100 ppm), our findings regarding conductivity are in agreement with the literature. Moghe [37] has shown a similar effect on the conductivity of the solution of polycaprolactone in glacial acetic acid. However, the viscosity of their solutions was increased and this was explained by the formation of large ionic complexes between pyridine and acetic acid. In our study, the viscosity has decreased significantly, which might be due to the fact that we investigated much lower concentrations of pyridine (between 1 to 100 ppm in volume to volume ratio) compared to Moghe and co-authors, who not only worked at the concentration of 1000 ppm and higher but also utilized different solvent which could react and form large ionic complexes with pyridine. In another study, Zeng and co-authors [38] showed that the conductivity of solutions of poly-L-lactide dissolved in dichloromethane was dramatically increased by adding pyridinium formiate (range: 2000 ppm– 8000 ppm), a salt form of pyridine, while it did not affect viscosity.

Similar results were reported by Huang et al. [34], who electrospun nylon-4,6 dissolved in formic acid with the addition of pyridine (concentrations above 370 ppm by weight). They showed that the addition of pyridine significantly changed the conductivity, with a negligible effect on the viscosity of the polymer solution.

### Pyridine increases the fibre deposition length and leads to smaller fibre diameters

Remarkably, the effect of pyridine on the deposition length was seen for concentrations as low as 1 ppm, i.e. at concentrations of two order of magnitudes lower than has previously been reported. The increase in deposition length shows a strong positive correlation with the increase in the solution’s conductivity and with the decrease of the electrospinning jet. This was expected as increasing the conductivity of the solution typically increases the jet bending instability [39, 40], resulting in a shortened straight jet, more whipping and ultimately in increased deposition length on the wire.

Similar results were observed when 0 ppm and 100 ppm PDO polymer solutions were spun onto a flat surface collector, as shown in S1 Fig.

External factors (level of cleanliness, temperature, type of materials used in electrospinning equipment, chemicals purity) also affect the electrospinning process and deposition length (or area). Here, the conductivity of polymer solution increased dramatically with very low concentration of pyridine (1 ppm). This suggests that impurities present in reagents used for electrospinning could affect the process. Such changes can occur when replacing research grade reagents by medical grade ones in the context of the clinical translation.

The combination of increased polymer solution’s conductivity, shortened jet and increased deposition length led to electrospun fibres with smaller diameters and with a more narrow fibre diameter distribution. This observation suggest that pyridine can also be used to tune the fibre diameter to a desired value. This is again consistent with the literature, since an increase in conductivity typically results in further thinning of the electrospinning jet due to stronger repulsions between electrical charges in the fluid resulting in thinner and more homogenous fibres [39, 41]. Previous researchers have already introduced the use of pyridine to achieve lower fibre diameter and resolve beading issues [34, 37, 38], however our investigation proves that similar effect can be achieved with much lower concentrations of pyridine.

### Filament usability

Improved quality of electrospun filaments was one of the aims of this study, as high quality product and robust manufacturing technology are essential in a mass manufacture of medical device and its compliance with associated regulations. Robust fabrication technology makes the upscale easier and adaptable to changes. Additionally, improved quality of the product leads to reduced wastage and increased process efficiency. In our study, this was achieved by the addition of pyridine which appeared to be a crucial step enabling the upscale of the aforementioned technology from the research setup to GMP manufacture, which is required to manufacture medical devices.

The improvement in filament fabrication process is reflected by the increased deposition length, which in our experience leads to better quality filaments. Although this assessment remains mostly qualitative so far, future work will look into quantitative assessment, such as counting the number of breaks per unit of length of filament.

### Pyridine decreases the drawing ratio and the strains at break of the filaments

The drawing step improves the mechanical performance of polymeric materials by removing their unfavorable ability to deform irreversibly during their use. There are number of parameters which can affect the drawing process, including material morphology, drawing ratio, speed of drawing, the temperature at which the process is performed and moisture content of the fibre [42, 43]. In this study, drawing at room temperature (or cold drawing) was applied.

The drawing ratio reflected the filaments strain at break which was inversely proportional to the concentration of pyridine. The decrease in strain at break prior to drawing correlates with the larger deposition area and the progressively decreasing fibre diameter, which results in more interconnected meshes.

Under cold drawing, the filaments were typically deformed non-homogenously by thinning down along their length in certain regions, creating necks. These then first deepened and lengthened to eventually grow in length without further change to depth [44]. The neck formation, both at the filament (macro) and the fibre (micro) levels, has been observed in our study and is described further in S2C Fig. It is known that the neck formation is accompanied by the alignment of the molecular chains in neck regions, which ultimately affected mechanical properties of drawn filaments [45, 46]. These interesting observations will be explored in future work.

### Pyridine increases the strength of filament

The addition of pyridine to PDO polymer solutions increased the strength of electrospun filaments. As for the strain at break, the increase in strength of the filaments comes from their morphology and is probably due to the more interconnected mesh that is created as the fibre diameter decreased.

It is interesting to note that the filament strength was further improved by the drawing step compared to undrawn filaments. A similar trend has been reported in our previous work [16] and by groups working with monofilaments produced by extrusion [7, 47–49] and for electrospun materials [50]. The increase in filaments strength is likely to be related to the molecular chain alignment occurring during drawing. Similar observations have been reported in literature [43, 50].

### Pyridine and medical devices

Pyridine is widely used in pharmaceutical, agricultural, food and other industries (as a solvent or intermediate in manufacture of pesticides, dyes, textile water repellents) [32, 33, 51]. Due to its toxicity, concerns regarding its safety have been raised, however, pyridine has not been classified as a carcinogenic to humans [31, 51]. In our study, pyridine was added to polymer solution in low concentrations as a controlled impurity in order to improve robustness of the manufacturing process. A measurement of residual pyridine in electrospun filaments is beyond the scope of this publication, however it is a part of biological evaluation required for implantable and absorbable medical device. As with any new medical device, the biological evaluation of the device and material constituents must be carefully assessed. Polydioxanone—the polymer used for filament manufacture, was selected on the basis of history of safe clinical use [17, 52, 53]. Addition of pyridine into the polymer solution must be carefully considered within the framework of the wider biological evaluation, which is guided by ‘ISO 10993 Biological evaluation of medical devices’ and a risk based approach must be adopted to the use of any additional raw materials (including solvents and controlled impurities). Further testing of the biological evaluation of the implantable medical device includes, but is not limited to: targeted chemical characterization, residuals testing, cytotoxicity testing, testing for local effects after implantation and systemic toxicity.

## Limitations

There are several limitations to be considered in this study. Firstly, in this study we focused on one additive compound–pyridine, while there are other means to increase solutions conductivity, such as the use of salts [54–56]. Future work will be carried out to see if similar observations are made with other compounds. Secondly, only a small range (1–100 ppm) of pyridine concentrations was investigated. While investigating the properties of filaments made with higher concentrations could be interesting, we have focused on using low concentrations to minimize the possibility for residual pyridine in the filaments designed for medical applications.

Finally, it is worth mentioning that this study only looked at one set of materials, PDO and HFIP. We believe that the findings presented in this article can be applied more generally to a wider combinations of polymers and solvents.

## Conclusions

The aim of this study was to improve the reliability of continuous electrospun filament fabrication by increasing the deposition length of the filament on the wire collector. We selected pyridine as a means to increase the conductivity of polymer solutions being used for electrospinning. We worked with low concentrations of pyridine, as from the biological risk assessment perspective these are favorable for medical yarn fabrication. To our knowledge, this is the first research in the electrospinning field that investigated the effect of pyridine at concentrations below 100 ppm.

We demonstrated that pyridine increased polymer solution conductivity at concentration as low as 1 ppm which was already sufficient to increase deposition length of fibres on the wire collector and to enable fabrication of usable filaments, which was not possible without adding pyridine to the polymer solution. We have also shown that pyridine addition at low concentrations (up to 100 ppm) changes the electrospun fibre diameter and the mechanical properties of the filaments.

Overall, this work suggests that the addition of pyridine could become a useful controlled impurity to both improve and fine-tune the fabrication of continuous electrospun filaments for medical applications.

## Supporting information

S1 Fig **Deposition area obtained in electrospinning experiment performed with two polydioxanone polymer solutions containing different concentrations of pyridine– 0 ppm (A) and 100 ppm (B)**. Both experiments were performed with a flow rate of 0.8 ml/h and the distance between charged nozzle and the grounded flat collector was 20 cm. Fibres were spun over 1 minute. Diameter of a deposition area was measured with a ruler.
The control (0 ppm) solution resulted in a jet length of around 6 cm and diameter of deposition area was 1 cm (the voltage applied was 11.7 kV). 100 ppm solution resulted in a jet length of around 2 cm and diameter of deposition area was 7 cm (the voltage applied was 10.7 kV).
(TIF)

S2 Fig Drawing of electrospun filament and elongation-force graphs analysis. (A) Determination of the Transition Point (TP) and of the EL_75_ (75% of the elongation between the TP and elongation at break). Two stretching ratios—DR_50_ and DR_75_, were tested in order to check if any unstretched sections remained in the filament which underwent drawing. DR_50_ resulted in unstretched sections detected within the drawn filament, whereas the drawing ratio established in 75% of the length between the transition and breakpoint resulted in fully stretched, but not broken filaments. Due to the sensitivity of our electrospinning setup, and therefore the variation in mechanical properties between filaments from different batches, the drawing ratio was calculated for an individual batch. Following equations were used:
Establishing EL_75_ for individual filament: EL_75_ = (0.75 x (E – TP)) + TP
Establishing drawing ratio: *L* = *L*_0_ + *EL*_75_; *DR*_75_ = *L*/*L*_0_
*EL*_*75*_*−75% of the elongation between the TP and elongation at break*. *Average EL*_*75*_ *was calculated for the batch (mm)*
*TP*–*Average Transition Point calculated for the batch*, *calculation based on three operators’ assessment (mm)*
*E*–*Elongation at break of the filament (mm)*
*L*–*Length of the stretched filament (mm)*
*L*_*0*_ *–Initial length of the filament (= 50 mm)*
(B) micro-level changes occurring in filament undergoing drawing–randomly oriented fibres are rearranged and become aligned. Necks are occurring mostly around the Transition Point. (C) Scanning Electron Microscopy (SEM) image of neck formation in partially stretched 5 ppm filament (magnification 200x). Necks were formed on electrospun filaments (“macro-neck”) but also on individual electrospun fibres (“micro-neck”) around the area of “macro-neck”. Magnified SEM pictures (7500x) show random fibres outside of the “macro-neck” (unstretched section) and more aligned in the area of “macro-neck” (stretched section). Necks occurring in individual electrospun fibres are indicated by arrows.
(TIF)
