# Supplementary figures and images for: Pyridine as an additive to improve the deposition of continuous electrospun filaments
Source: PLoS One. 2019 Apr 25;14(4):e0214419. doi: 10.1371/journal.pone.0214419 (PMC6483168; doi:10.1371/journal.pone.0214419)

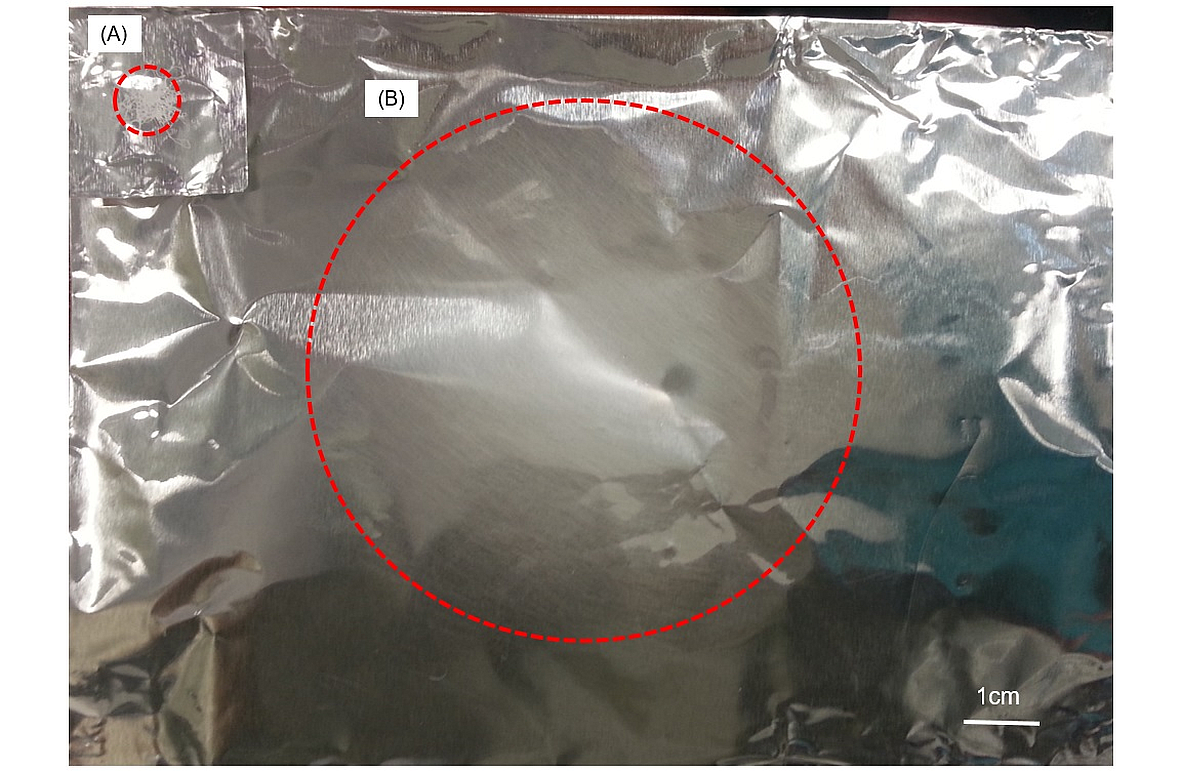

Supplement: S1 Fig — Deposition area obtained in electrospinning experiment performed with two polydioxanone polymer solutions containing different concentrations of pyridine– 0 ppm (A) and 100 ppm (B). Both experiments were performed with a flow rate of 0.8 ml/h and the distance between charged nozzle and the grounded flat collector was 20 cm. Fibres were spun over 1 minute. Diameter of a deposition area was measured with a ruler. The control (0 ppm) solution resulted in a jet length of around 6 cm and diameter of deposition area was 1 cm (the voltage applied was 11.7 kV). 100 ppm solution resulted in a jet length of around 2 cm and diameter of deposition area was 7 cm (the voltage applied was 10.7 kV). (TIF) [file pone.0214419.s001.tif]

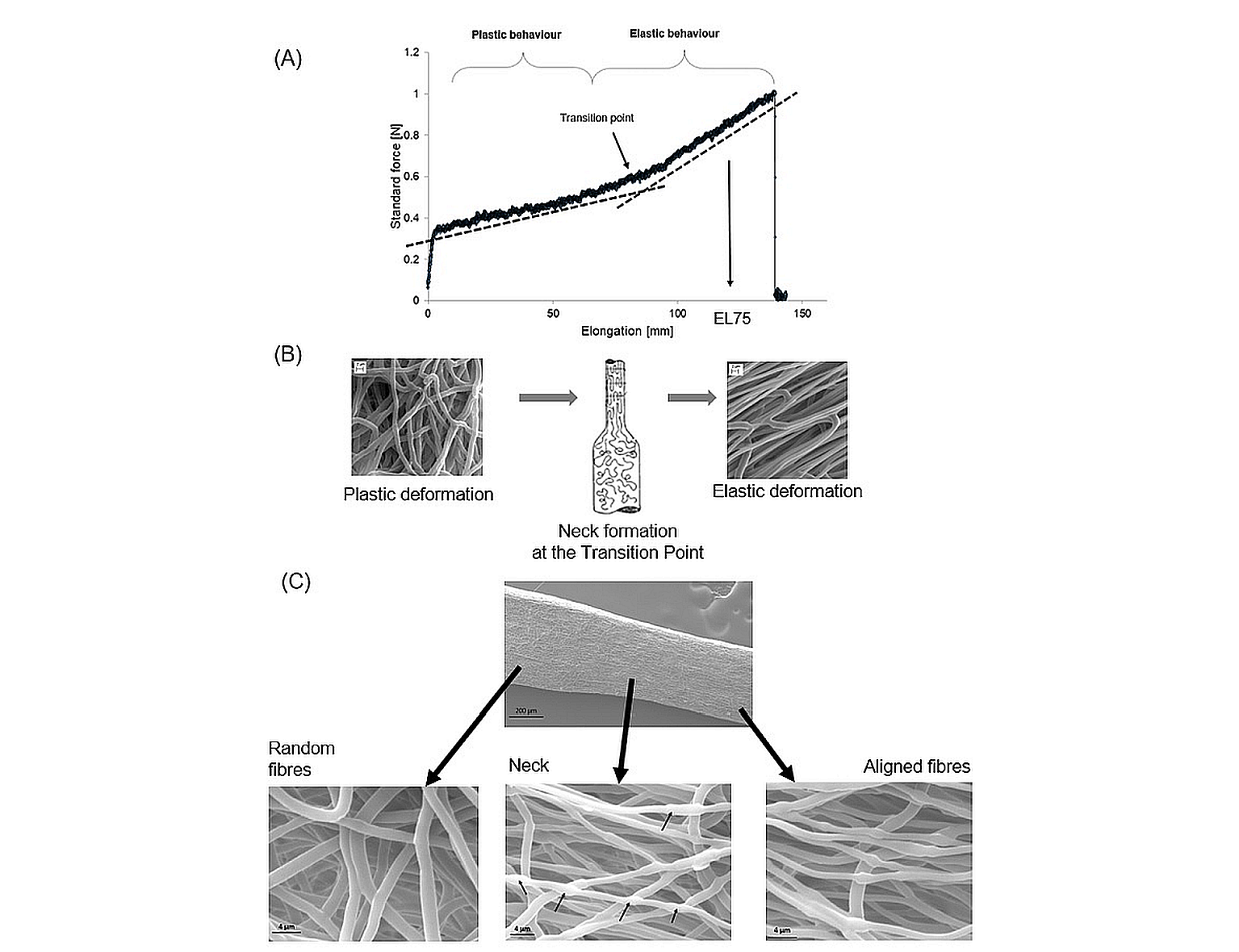

Supplement: S2 Fig — (A) Determination of the Transition Point (TP) and of the EL75 (75% of the elongation between the TP and elongation at break). Two stretching ratios—DR50 and DR75, were tested in order to check if any unstretched sections remained in the filament which underwent drawing. DR50 resulted in unstretched sections detected within the drawn filament, whereas the drawing ratio established in 75% of the length between the transition and breakpoint resulted in fully stretched, but not broken filaments. Due to the sensitivity of our electrospinning setup, and therefore the variation in mechanical properties between filaments from different batches, the drawing ratio was calculated for an individual batch. Following equations were used: Establishing EL75 for individual filament: EL75 = (0.75 x (E – TP)) + TP Establishing drawing ratio: L = L0 + EL75; DR75 = L/L0 EL75−75% of the elongation between the TP and elongation at break. Average EL75 was calculated for the batch (mm) TP–Average Transition Point calculated for the batch, calculation based on three operators’ assessment (mm) E–Elongation at break of the filament (mm) L–Length of the stretched filament (mm) L0 –Initial length of the filament (= 50 mm) (B) micro-level changes occurring in filament undergoing drawing–randomly oriented fibres are rearranged and become aligned. Necks are occurring mostly around the Transition Point. (C) Scanning Electron Microscopy (SEM) image of neck formation in partially stretched 5 ppm filament (magnification 200x). Necks were formed on electrospun filaments (“macro-neck”) but also on individual electrospun fibres (“micro-neck”) around the area of “macro-neck”. Magnified SEM pictures (7500x) show random fibres outside of the “macro-neck” (unstretched section) and more aligned in the area of “macro-neck” (stretched section). Necks occurring in individual electrospun fibres are indicated by arrows. (TIF) [file pone.0214419.s002.tif]
